# Combining Zebrafish and CRISPR/Cas9: Toward a More Efficient Drug Discovery Pipeline

**DOI:** 10.3389/fphar.2018.00703

**Published:** 2018-07-03

**Authors:** Carles Cornet, Vincenzo Di Donato, Javier Terriente

**Affiliations:** ZeClinics SL, PRBB (Barcelona Biomedical Research Park), Barcelona, Spain

**Keywords:** CRISPR/Cas9, drug discovery, zebrafish, disease model, phenotypic drug screening, functional genomics

## Abstract

The use of zebrafish larvae in basic and applied research has grown exponentially during the last 20 years. The reasons for this success lay in its specific experimental advantages: on the one hand, the small size, the large number of progeny and the fast life cycle greatly facilitate large-scale approaches while maintaining 3Rs amenability; on the other hand, high genetic and physiological homology with humans and ease of genetic manipulation make zebrafish larvae a highly robust model for understanding human disease. Together, these advantages allow using zebrafish larvae for performing high-throughput research, both in terms of chemical and genetic phenotypic screenings. Therefore, the zebrafish larva as an animal model is placed between more reductionist *in vitro* high-throughput screenings and informative but low-throughput preclinical assays using mammals. However, despite its biological advantages and growing translational validation, zebrafish remains scarcely used in current drug discovery pipelines. In a context in which the pharmaceutical industry is facing a productivity crisis in bringing new drugs to the market, the combined advantages of zebrafish and the CRISPR/Cas9 system, the most powerful technology for genomic editing to date, has the potential to become a valuable tool for streamlining the generation of models mimicking human disease, the validation of novel drug targets and the discovery of new therapeutics. This review will focus on the most recent advances on CRISPR/Cas9 implementation in zebrafish and all their potential uses in biomedical research and drug discovery.

## A Productivity Crisis in the Pharmaceutical Industry

During the last decades, the ratio between the number of new therapeutic drugs (NTD) reaching the market and R&D expenditure has suffered an important decrease (Paul et al., 2010; Scannell et al., 2012). Thus, despite the creation and rapid growth rate of dozens of biotechnological companies and important merges and acquisitions, the biopharmaceutical industry is suffering a productivity crisis. This crisis is mostly explained by the extremely high rate of drug attrition for molecules entering clinical trials (Kola and Landis, 2004), in which 95% of the compounds fail after clinical phases II and III. Mayor sources of drug attrition are lack of efficacy, accounting for 50%, and attrition due to safety liabilities, accounting for 25% (Waring et al., 2015). This massive attrition rate results in an average cost for bringing an NTD to the market of $2.5 billions (DiMasi et al., 2016). Such economic burden hinders the industry progression and limits its commitment for facing diseases in which the return of investment (ROI) is not well-defined, such as rare, third world or complex first world diseases (Schmid and Smith, 2007). A recent example is Pfizer’s decision to interrupt their research programs on Alzheimer’s and Parkinson disease (Reuters.com, 2018). There are two main elements to consider regarding low clinical approval rate: (i) how predictive is the preclinical phase toward human safety and efficacy, and (ii) how appropriately are the drug targets chosen to tackle a particular disease.

Regarding the predictivity of the preclinical phase, it is worth pointing out that all drugs that fail in clinical phases have gone through a supposedly comprehensive preclinical phase. Hence, the low NTD acceptance rate suggests that the informations gathered during preclinical phases, specifically that obtained from animal models, provides lower-than-expected prediction of toxic liabilities and therapeutic effects in human patients (Scannell and Bosley, 2016). Therefore, it is necessary to develop strategies that improve the predictive value of current preclinical animal models and/or combine them with better *in silico* and *in vitro* tools in order to narrow down the most promising candidates before entering expensive preclinical and clinical phases. On the subject of how drug targets are chosen, it has become apparent that clinical success increases with a deeper understanding of a disease and its related biological pathways. Thus, drugs which modulate targets directly associated with the pathology show a higher success rate in both preclinical and clinical phases (Nelson et al., 2015). Unfortunately, identifying genetic-disease associations is not an easy task and it might not even lead to the discovery of an appropriate druggable target. A paradigm is the *fat mass and obesity-associated protein* (*FTO*) gene: as the name indicates, single nucleotide polymorphisms (SNPs) identified in this gene have been associated with obesity and type 2 diabetes risk (Loos and Yeo, 2014). In this case, the association between gene and pathology remains undisputed; however, it has been shown that SNPs identified in *FTO* introns 1 and 2 were actually associated with the long-range positive regulation of *IRX3* in the human brain. Interestingly, *IRX3* overexpression had a clear impact in weight gain in animal models and a clear correlation with the expression data obtained from obese patient samples (Smemo et al., 2014). The exact number of drug discovery initiatives targeting *FTO* to treat obesity or type 2 diabetes is unknown to us, but the findings of Smemo et al. (2014)– obtained by combining experimental data from several animal models including zebrafish – illustrate the need of carrying out detailed genetic functional studies (i.e., functional genomics) before entering costly drug discovery programs. All in all, decreased productivity and high drug attrition, either due to low preclinical predictivity or poorly chosen targets, highlights the need of innovative strategies to streamline the drug discovery pipeline (Plenge, 2016).

## Zebrafish Research and Biomedical Applications

### Zebrafish: From Basic Research to Drug Discovery

Zebrafish is a small fresh water fish that has been used for decades as a classical developmental biology research model (Streisinger et al., 1981; Kimmel, 1989). Its use increased exponentially from the 1990s’, when several genetic screens showed the potential of this animal model in identifying and characterizing novel genes involved in vertebrate development and disease. The zebrafish specific characteristics such as the large number of progeny and external development of the larvae, fast life cycle, small size and transparency allowed performing large-scale genetic screenings, which would have been unattainable in mammalian models (Driever et al., 1994; Haffter and Nüsslein-Volhard, 1996; Lawson and Wolfe, 2011). Such screens followed the example of previous studies performed with *Caenorhabditis elegans* and Drosophila (Brenner, 1974; Nusslein-Volhard and Wieschaus, 1980), but were revolutionary on their own, given that a vertebrate model allowed the identification and validation of genes in a context closer to human biology than that provided by invertebrate genetic models. Nowadays, research in zebrafish has expanded from basic research toward most translational biomedical areas. Three additional features have fuelled that transition: First, ∼83% of human disease-related genes have functional orthologs in zebrafish (Howe et al., 2013), suggesting that human pathologies can be faithfully modeled in zebrafish. Indeed, that has been the case for several indications such as cancer (Terriente and Pujades, 2013; White et al., 2013), cardiovascular (Asnani and Peterson, 2014) or neurologic diseases (Clark et al., 2011). Second, liver, kidney, and tissue barriers are functional from early development (Parng, 2005). Therefore, zebrafish physiology recapitulates mammalian drug metabolism features – Absorption, Distribution, Metabolism and Excretion (ADME) – and provides *de facto* a body-on-chip experimental set up. Third, zebrafish larvae are not considered animals by animal welfare regulation before 5 days post fertilization (dpf), a stage when they start independent feeding. Hence, using zebrafish larvae in research has a direct impact in the Replacement, Reduction, and Refinement (3Rs) of animal models, which is a crucial aspect for raising the ethical standards in the pharmaceutical and chemical industry (Avey et al., 2015). These facts suggest that a broader use of zebrafish could benefit the biomedical community in streamlining the drug discovery process. In that sense, regulatory agencies recommend the use of this and other small animals (FDA, 2004). However, before being fully adopted by regulatory agencies and the pharmaceutical industry, drug discovery through zebrafish might require better validation and a deeper understanding on biologic translatability toward humans. To advance on the validation front, several studies have focused on addressing how precise is the correlation of drug activity between zebrafish and human gathered data (Milan et al., 2003; Ali et al., 2011; Cornet et al., 2017). Those studies show how using zebrafish predicts toxicity liabilities for more than 80% of the drugs. Regarding biologic translatability, we stated above the high conservation in genes, protein structure and physiology with humans. However, to further prove the applicability of the zebrafish model during the drug discovery process, an important step would be the development of humanized zebrafish models, in which native genes would be exchanged by their human orthologues, therefore, recapitulating same biological pathways but with an intact human target protein structure. This feature would indeed provide more solid drug-target interaction evidences. Either way, the collective efforts from the zebrafish research community will be required to fully overcome these “validation” and “translatability” challenges. In the meantime, we will discuss below some of the hallmarks and general advantages achieved by using zebrafish in drug discovery today.

### Zebrafish: Speeding up the Drug Discovery Pipeline

Traditionally, the pharmaceutical industry has used two main strategies to discover new drugs: target-based drug screenings, in which drugs are identified *in vitro* based on their binding properties to specific molecular targets (e.g., recombinant proteins), and phenotypic drug screenings, in which drugs are identified, *in vitro* or *in vivo*, based on the modification of a disease phenotype in cells, tissues or whole organisms. Determining the relevant drug target/s identified through phenotypic screening was often slow and sometimes impossible. That fact tilted the pharmaceutical industry efforts toward target-based screenings. However, these strategies have demonstrated lower drug discovery success rates than phenotypic drug screenings (Swinney and Anthony, 2011). Nowadays, innovation on *in silico* and *in vitro* target identification tools allows a faster and more precise determination of molecular targets (Schenone et al., 2013; Cereto-Massagué et al., 2015), which is positioning phenotypic drug discovery back in trend (Kotz, 2012).

Despite some challenges stated above, zebrafish is a very suitable and reliable experimental model for performing phenotypic drug discovery. In fact, the use of zebrafish is already helping the pharmaceutical industry on three different fronts. First, by validating potential druggable targets identified through genomic screenings on human patient populations (Liu et al., 2013); second, by generating novel disease models to better understand pathogenesis (Ablain and Zon, 2013); and third, by using those disease models, or other biological features, as the basis for performing phenotypic drug screenings designed to identify new therapies (MacRae and Peterson, 2015). Some examples are: Proto-1, which protects against toxicity in ciliated cells of the inner ear caused by antibiotics (Coffin et al., 2010); inhibitors for PDE5A to treat Duchenne Muscular Dystrophy (Kawahara et al., 2011), which are currently in clinical phases; or Dorsomorphin, a BMP inhibitor applicable in the treatment of progressive ossifying fibro dysplasia (Yu et al., 2008).

The implementation of CRISPR/Cas9 technology, a straightforward (Hwang et al., 2013), in zebrafish and precise genome editing technique, is streamlining the process for achieving better disease modeling, target validation and drug discovery.

## CRISPR/Cas9 in Zebrafish

Several methods have been developed and applied in zebrafish to alter gene transcription and function (Koster and Sassen, 2015). Among them, CRISPR/Cas9, a system that allows rapid and accurate genome editing, has become the most widespread technique in zebrafish and other model systems. The CRISPR/Cas9 experimental basics and general applications have been reviewed extensively before (Hsu et al., 2014; Barrangou and Doudna, 2016; Fellmann et al., 2017). However, it is important to reiterate some important details relevant to this review. All gene editing methods, including CRISPR/Cas9, are based on the inherent capability of cells to repair their genome after DNA Double Strand Break (DSB) events (Chang et al., 2017). DNA repair relies, in part, on the Non-Homologous End Joining (NHEJ) mechanism, a homology-independent error-prone pathway promoting, in a variable percentage, the appearance of *de novo* insertions/deletions (INDELs). NHEJ can result into the disruption of a coding sequence or regulatory region and, therefore, the inactivation of a gene of interest (NHEJ-mediated knockout). Additionally, NHEJ can be exploited to insert exogenous DNA fragments, such as reporters or drivers in the genome (NHEJ-mediated knockin). Alternatively, knockin of DNA fragments can be performed through a different DNA repair pathway: Homology Directed Repair (HDR). This pathway requires the availability of a homologous DNA template to promote DNA repair through Homologous Recombination (HR). Several applications have been developed *via* HDR (HDR-mediated knockin) to achieve precise, programmed modification of the zebrafish genome: introduction of point mutations to mimic specific human SNPs and/or integration of LoxP sites for site specific recombination or fluorescent reporters. However, in zebrafish and other systems, HDR is still a challenging approach due to the low rate of DSB repair by HR compared to NHEJ (Maruyama et al., 2015; Horii and Hatada, 2016). An additional strategy is the use of modified Cas9 proteins, which do not cleave DNA but allow generating SNP exchange or regulate transcription. Below, we will discuss all these methodologies, their challenges and potential biomedical applications toward the discovery of new therapies for humans.

### CRISPR/CAS9-Mediated Knockout

Induced mutagenesis of genes of interest in zebrafish can be achieved with a relatively straightforward experimental setup. The method displaying the highest mutagenesis efficiency is based on microinjection of an *in vitro* pre-assembled complex of guide RNA and Cas9 protein in one-cell stage embryos. There are two possible strategies after F0 animals have been injected. Either, F0 injected larvae, carrying mosaic loss-of-function (LOF) mutations (INDELs), can be directly phenotyped and used to study the function of candidate genes, a strategy known as transient knockout approach. Or, F0 larvae can be grown to sexual maturity and crossed to generate F1 heterozygous carriers and F2 homozygous mutant larvae, a so-called isogenic stable knockout. The generation of an isogenic stable knockout takes 6 months and allows obtaining hundreds of F2 larvae (homozygous, heterozygous and wild type siblings), which can be used to prove a research hypothesis or to evaluate in parallel several therapeutic drug candidates in a robust biological background.

#### Transient Knockout

The advent of next-generation sequencing has contributed to the identification of a growing number of candidate genes potentially associated with human disease. To tackle this considerable amount of data, a high-throughput strategy for validating candidate genes phenotypically would be very advantageous. Along this line, a report showed the mutagenesis of 83 genes (162 loci) with a 99% success rate, and an average germline transmission rate of 28%. It also showed that by inbreeding two founder fish, phenotyping can be performed in the *F1* generation, resulting in a significative reduction time and space required for animal husbandry (Varshney et al., 2015). Another high-throughput CRISPR-Cas9 phenotyping screen, targeting 48 genomic loci, identified two genes involved in electrical synapse formation (Shah et al., 2015). Due to the high efficiency of somatic mutation, the authors were able to detect specific phenotypes already in injected F0 animals. In a more recent report, the *in vitro* assembly optimization of Cas9 and sgRNA riboprotein complexes (RNPs) allowed the generation of so-called Crispants (CRISPR/Cas9-mediated mutants), which yields high rates (up to 100%) of somatic mutagenesis upon injection. Indeed, this report shows full penetrance of phenotypes, such as pigmentation defects or heart edema, by targeting several genomic loci and recapitulating, in injected F0 larvae, LOF phenotypes displayed in homozygous isogenic mutants (Burger et al., 2016). Similarly, a recent report showed how the simultaneous injection of different sgRNAs targeting the same allele could promote up to 99% of somatic mutations. When this approach was tested on two genes from the KEOPS complex, transient injected larvae displayed the same microcephaly and low survival phenotypes previously observed in isogenic homozygous larvae (Jobst-schwan et al., 2018).

Regarding the challenges of this application, it is important to note that CRISPR/Cas9 has been suggested to produce false-negative results due to genetic compensation (Rossi et al., 2015). This limitation should be considered when validating potential drug targets during the drug discovery process. Another evident drawback of this approach could be low somatic penetrance and mosaicism, which can result in contiguous cells being wild type and mutant or different animals showing a variable phenotypic degree. To counteract this issue, fast and accurate genotyping tools, such as IDAA^TM^ (Lonowski et al., 2017) and TIDE (Brinkman et al., 2014), allow to perform quantitative correlation between mutagenesis rate and phenotype penetrance in single individuals. All in all, this somatic mosaic knockout approach allows the phenotypic screening of genes and pathways, providing a fast method for performing target validation for disease-relevant genes identified through genomic strategies. However, a transient approach does not provide the phenotypic robustness provided by the use of isogenic mutant lines explained below.

#### Isogenic Stable Knockout

Many zebrafish mutant models have been developed through CRISPR/Cas9 (Liu et al., 2017). Successful models include neurological, kidney, hepatic, cardiovascular, muscle/skeletal or structural birth defects such as orofacial clefts and heterotaxy (Chang et al., 2013; Borck et al., 2015; Bolar et al., 2016; Noël et al., 2016; Duncan et al., 2017; Ellis et al., 2017; Küry et al., 2017; Shaw et al., 2017; Van De Weghe et al., 2017; Zabinyakov et al., 2017). A paradigm of the exploitation of zebrafish disease modeling through CRISPR/Cas9 is found in the development of a zebrafish line carrying a LOF mutation in the ribosomal protein S14 gene (*rps14*). This model was generated to understand the effect of RPS14 deficiency in the 5q-deletion Syndrome (Ear et al., 2016). 5q-syndrome is a form of myelodysplastic syndrome (MDS) characterized by bone marrow failures, including macrocytic anemia. *rps14* zebrafish mutant displayed gross morphological defects accompanied with an elevation in p53 activity. Furthermore, an anemic phenotype, typically seen in patients with disrupted ribosome gene function, was identified in fish carrying LOF alleles. Interestingly, those phenotypes were rescued through treatment with RAP-011, L-leucine, and dexamethasone. These are promising results for future clinical trials, since two of these small molecules have a p53-independent mechanism of action. Therefore, they represent a valuable alternative to therapeutic treatments targeting p53 for patients with ribosomopathies, which have high incidence of later cancer development. (See **Table 1** for a summary of the aforementioned disease models).

**Table 1:** Protein/gene targeted through CRISPR/Cas9, the disease/phenotype studied and the corresponding reference.

Protein/gene targeted	Disease/phenotype studied	Reference
*osgep* and *tprkb*	Microcephaly and reduced survival	Jobst-schwan et al., 2018
*sec61al2*	Autosomal-dominant tubulo-interstitial kidney disease	Bolar et al., 2016
*brf1*	Cerebellar-dental-skeletal syndrome	Borck et al., 2015
*etsrp* and *gata5*	Vessel phenotypes (*etsrp*) Cardia bifida phenotypes (g*ata5*)	Chang et al., 2013
*abcb11b*	Progressive familial intrahepatic cholestasis type 2	Ellis et al., 2017
*cfap53*	Dextrocardia and heterotaxy (left-right asymmetry)	Noël et al., 2016
*psmd12*	Syndromic neurodevelopmental disorder	Küry et al., 2017
*smchd1*	Bosma arhinia microphthalmia syndrome	Shaw et al., 2017
*armc9*	Ciliopathy phenotypes	Van De Weghe et al., 2017
*aldh7a1*	Pyridoxine-dependent epilepsy	Zabinyakov et al., 2017
*rps14*	5q-deletion Syndrome	Ear et al., 2016
*urod*	Human hepatic cutaneous porphyria	Ablain et al., 2015
*sox10*	Melanoma	Kaufman et al., 2016
*insra/insrab*	Hepatic dysfunction	Yin et al., 2015
*tardbp* and *fus*	Amyotrophic lateral sclerosis	Armstrong et al., 2016
*twist2*	Ablepharon macrostomia syndrome	Zhang et al., 2016, 2017
*pax2a*	Loss of the midbrain-hindbrain boundary	Ota et al., 2016
*tyr*	Oculocutaneous albinism	Zhang et al., 2017
*pitx2*	Ocular dysgenesis	Protas et al., 2017

Despite the experimental time required for isogenic mutant isolation, phenotypic validation and use of the generated model in drug discovery, disease modeling in zebrafish represents a valuable approach – considering time and cost saving – to analyze the pathogenic effect of a given mutation or test a battery of candidate drugs before proceeding to further preclinical trials with mammalian animal models. In fact, efficacy information gathered through zebrafish could be enough for advancing toward clinical phases, if provided together with the required toxicity profile obtained in regulatory animals.

#### Tissue-Specific Knockout

Gene knockout may result in embryonic lethality when targeted genes are involved in crucial developmental or housekeeping activities. This represents a limitation for the phenotypic analysis of disease-causing mutations, especially when the readout is expected to be tissue-specific. To overcome such limitations, different conditional knockout methods have been developed in zebrafish. The first study described a CRISPR-based vector system for tissue-specific gene inactivation, based on the tissue-specific expression of Cas9 and ubiquitous expression of a single sgRNA targeting a gene of interest. In detail, the erythrocyte-specific *gata1* promoter drove *Cas9* expression and *urod*, which is implicated in heme biosynthesis, was the chosen target gene (Ablain et al., 2015). Mutations in the *UROD* gene are found in human hepatic cutaneous porphyria, a disorder characterized by defects in iron metabolism in liver, skin photosensitivity and reduced erythrocytic heme production (Balwani and Desnick, 2012). Furthermore, *urod*-deficient erythrocytes exhibit strong red fluorescence due to the accumulation of unprocessed porphyrins, which are inherently fluorescent. In zebrafish, it was found that *urod* inactivation in erythrocytes led to the appearance of fluorescent erythrocytes at 30 hpf, mimicking the phenotype seen in humans and in *yquem* mutants, an additional *urod* mutant described before (Wang et al., 1998). A similar approach allowed the genetic inactivation of *sox10* in melanocytes to study its role in melanoma initiation. In this case, zebrafish embryos were injected with a vector expressing *Cas9* under the control of the melanocyte-specific *mitfa* promoter and an sgRNA targeting *sox10* (Kaufman et al., 2016). A third report showed the development of a double transgenic approach. On one hand, *Cas9* was expressed either ubiquitously or in a tissue-specific manner. Cas9 lines were combined with transgenic lines expressing up to five sgRNAs under the control of different U6 promoters. With this strategy, a fish model for hepatic dysfunction caused by altered glucose homeostasis was developed through the liver-specific abolishment of insulin signaling (Yin et al., 2015) (see **Table 1** for a summary). In another study, Di Donato et al. (2016) expanded the tissue-specific gene disruption toolbox by combining CRISPR/Cas9 and Gal4/UAS systems. To this end, a vector system called 2C-Cas9 (Cre-mediated recombination for Clonal analysis of Cas9 mutant cells) was developed, based on the UAS-driven expression of Cas9 and U6-driven expression of two different sgRNAs. UAS-driven expression of Cas9 offers the possibility of conditional targeted mutagenesis in virtually any cell-type through the use of the broad repertoire of available tissue-specific zebrafish transgenic Gal4 driver lines (Di Donato et al., 2016; Albadri et al., 2017a).

These methods offer the possibility to study gene function in specific tissues. Moreover, optimization of these tools should allow simultaneous gene inactivation and mutant cell fate analysis through fluorescent cell tracing. This last characteristic addresses a crucial issue in the analysis of the effects of gene inactivation in model organisms: direct correlation between pathogenesis, genotype and cell/tissue phenotype. From a more translational point of view, these approaches would allow generating disease models, based on targeting specific tissues, for those genes that might promote embryonic lethality before the disease phenotype can be addressed.

### Knockin

Targeted insertion (knockin) of small or large DNA fragments is a promising, but for the moment not very widespread method to generate disease models in zebrafish. We will discuss knockin methodologies according to the DNA repair pathway they exploit to achieve DNA insertion: HDR or NHEJ. Additionally, we will discuss an alternative method for SNP exchange that does not rely on DSB repair.

#### HDR-Mediated Knockin

Homology directed repair allows the precise integration of DNA fragments. In general terms, small modifications such as single nucleotide editing or LoxP integration can be achieved by providing a single-stranded oligodeoxynucleotide (ssODN) as donor DNA (Chang et al., 2013) for CRISPR/Cas9-mediated HDR. By using this approach, Armstrong et al. (2016) generated a zebrafish model of amyotrophic lateral sclerosis (ALS), *via* insertion of two SNPs in the zebrafish *tardbp* and *fus* genes (*tardbp*^A379T^ and *fus*^R536H^, respectively), corresponding to *tardbp*^A382T^ and *fus*^R521H^ disease-causing point mutations identified in patients with ALS (Armstrong et al., 2016) (**Table 1**). Albeit this represents a rapid and straightforward approach for the knockin of point mutations of interest, the low efficiency of germline transmission of the mutation represents an important drawback (the maximum reported efficiency was only 4%). An increase in efficiency of HDR-mediated knockin of ssODN was recently reported (Moreno-Mateos et al., 2017). The authors made use of an alternative Cpf1 CRISPR/Cas DNA nuclease derived from *Lachnospiraceae bacterium ND2006*, LbCpf1, which proved to induce homology-directed integration of optimized single strand DNA donors four times more efficiently than Cas9.

For larger DNA sequences, the consensus is to use plasmids as donor DNA (Irion et al., 2014). By using this methodology, in a recent report the *twist2* gene was successfully targeted to mimic a human mutation found in Ablepharon macrostomia syndrome (AMS) (**Table 1**). Here, the authors made use of a double-strand long arm donor plasmid as template for HDR, with the total length of the inserted sequence being less than one kilobase (Kb), in order to induce precise nucleotide substitution (Zhang et al., 2016). In this case, the transmission of edited alleles in the germline could be detected in around 3% of the cases. For both types of DNA donors – ssODN or plasmids – the main drawback is the low efficiency of integration. To counteract low efficiency, it has been proposed that using NHEJ drug antagonists (scr7) or HDR drug agonists (RS-1) could increase HDR homologous recombination (Song et al., 2016). In our hands, both drug treatments have a low impact on HDR efficiency (data not shown). Nonetheless, a consistently higher frequency of germline transmission has been shown when CRISPR/Cas9 complex is co-injected with donor plasmids, where the DNA insert is placed between 1 Kb long homology arms flanked by I-SceI meganuclease restriction sites. This approach is different from other knockin methods in the use of long homology arms and the pre-digestion of the donor plasmid with I-SceI meganuclease (Hoshijima et al., 2016). Both features, together with achieving high rates of initial DSB through careful selection of highly efficient sgRNAs, might be determinant in increasing precise integration rate in zebrafish. In our opinion and that of others (Albadri et al., 2017b), methodologies based on the use of large homology arms are the most appropriate tool in order to generate HDR knockins for large or small modifications.

Regardless of the efficiency rate, HDR-based strategies remain the most accurate method for modification of targeted sequences. In that sense, results of HDR-based knockin strategies are promising and their potential applications in disease modeling and personalized medicine very broad. However, the efficiency of the available methods remains extremely low. That might explain the scarcity of disease models with precise modifications published through these methods. Indeed, further improvements and alternative strategies will have to be developed. In that respect, implementation of HDR-independent SNP exchange strategies might help to widen the disease model spectrum.

#### NHEJ-Mediated Knockin

As an alternative to methodologies based on low-efficient HDR mechanisms, targeted insertion of exogenous DNA fragments can be achieved by taking advantage of NHEJ repair after DSB events. Despite NHEJ being an error prone mechanism resulting in INDELs generation, it has also been shown to promote repair through the integration of donor DNA in a highly efficient fashion. In zebrafish, a pioneer work developed a homology-independent CRISPR/Cas9-mediated integration of reporter genes at defined target genomic loci. This approach is based on the concurrent cleavage of a donor vector and a targeted genomic sequence. It was first tested with a DNA donor containing a cassette coding for the transcriptional activator Gal4 and sgRNAs targeting the eGFP locus in transgenic zebrafish lines. Targeted integration of the Gal4 cassette successfully allowed converting eGFP transgenic lines into Gal4 drivers, significantly expanding the potential of Gal4-UAS technology in zebrafish. Interestingly, it was shown that even native zebrafish genes can successfully be targeted for integrating exogenous DNA, eGFP in this case (Auer et al., 2014). Since NHEJ-mediated knockin can take place in either possible orientation, this methodology has been improved by the addition of a heat shock promoter (*hsp70*) to the donor plasmid. This is intended for overcoming the need of in-frame insertion of the donor cassette to activate the reporter transgene (Kimura et al., 2014). This methodology has also been applied for integrating Cre-ER^T2^ recombinase into the *otx2* gene locus to generate a conditional Cre-driver line specific to the anterior neural plate (Kesavan et al., 2018). Additionally, it has been used for generating LOF alleles through the integration of GFP cDNA. In that study, inactivation of *pax2a* is achieved by integrating a donor plasmid containing an eGFP cassette. It is worth noting that, fish homozygous for the DNA cassette insertion not only display fluorescence in the expression domains of *pax2a*, but also recapitulate the phenotype observed in the well characterized *pax2a/noi* mutant consisting in loss of midbrain hindbrain boundary and aberrant projection of optic axons (Ota et al., 2016). This latter approach has the advantage of generating a mutant and a fluorescent reporter at once.

The main drawback of this methodology is that repair at the sites of DNA integration is often imprecise. Additionally, the donor vector is integrated as a whole. Hence, DNA integration is likely causing concomitant LOF on the target gene; an unintended side effect for some applications. Moreover, this technology cannot be applied when precise integration such as in protein tagging is required. Nonetheless, high efficiency and versatility are important advantages for reporter line generation and other applications, when compared to HDR approaches. From a translational point of view, the use of these methodologies could allow the generation of more precise reporter lines for several genes or signaling pathways. That could allow, for example, to identify drugs altering Notch, WNT, or BMP signaling, which are important players in development, but also in cancer progression (Terriente and Pujades, 2013).

#### DNA Base Editing

Recently, a strategy for precise single “base editing” (BE), developed in mammalian cells (Komor et al., 2016), has been implemented in zebrafish (Zhang et al., 2017). BE system is based on the fusion of a cytidine deaminase to a Cas9 nickase (nCas9), which allows a DSB-independent irreversible conversion of one targeted base to another. This methodology achieved the conversion of cytidine to thymine, adenine and guanine at different genomic loci mimicking causative mutations of human diseases such as AMS and oculocutaneous albinism (**Table 1**). The same report showed that it is possible to expand the number of potential genomic targets by replacing Cas9 nickase with a so called VQR variant nickase, which recognizes the 5′-NGA PAM. Importantly, germline transmission of targeted modifications ranged between 7 and 37%, making this approach a valuable alternative to lower efficiency HDR-mediated methods. Certainly, the use of base editors will allow the development of several zebrafish disease models mimicking specific human polymorphisms. Several improvements are expected. In particular, it is not yet possible to target all desired SNPs in zebrafish, due to specific limitations of base targeting – the binding of the nickase requires the presence of a PAM sequence adjacent to the targeted site; not every base can be converted in another. An advance in this direction has been provided by a recent report, in which the authors show the development of adenosine deaminase editors (ABEs) to allow efficient conversion of A-T into G-C base pairs; again without induction of DSBs (Gaudelli et al., 2017). A further implementation of these tools for genome engineering in zebrafish would greatly expand the current possibilities of studying human-associated polymorphisms *in vivo*.

As a challenge to these approaches, genotyping point mutations can be cumbersome. Unless specific restriction sites are created or destroyed by the point mutation, every single individual will need to be Sanger sequenced, which would certainly escalate time and cost of the whole procedure. These approaches would have the same translational applications – target validation and disease modeling – mentioned for KO animals.

### CRISPR-Based Transcriptional Regulation

Transcriptional regulation can be achieved by using another modified Cas9 protein lacking the catalytic endonuclease activity: Dead Cas9 (dCas9) (Qi et al., 2013). This Cas9 mutant form is still guided by sgRNAs and has been used to repress (CRISPRi) or activate (CRISPRa) gene transcription without introducing irreversible genomic mutations. The dCas9 protein can act on its own when targeted to the coding region of a gene by blocking transcription. When dCas9 is fused to a repressor domain such as KRAB or activator domain such as VP64, it can also interact with regulatory regions to either activate or repress transcription (Long et al., 2015). As an alternative to CRISPRi/a, the deletion or modification of conserved regulatory regions in the zebrafish genome could also help to understand the role of polymorphisms identified in non-coding regions, and how they are associated to human disease. This approach was used to identify genes associated to ocular dysgenesis. Here, a large genomic deletion upstream of *pitx2* in the genome of ocular dysgenesis patients was identified. The deletion contained several non-coding elements – potential enhancers – that are conserved in the zebrafish genome. In line with that, zebrafish larvae homozygous for deletions on those conserved regions displayed a similar ocular phenotype than human patients, which suggests a role of *pitx2* transcriptional regulation in the progression of ocular dysgenesis (Protas et al., 2017) (**Table 1**).

Approaches, which have a greater impact in gene regulation than in protein function could be used to screen rapidly both, loss-of-function and gain-of-function phenotypes, and such strategy, can provide complementary information to knockouts for mapping complex pathways. Moreover, this represents an alternative mean to further exploit genome wide association study (GWAS) data and to ultimately identify polymorphisms situated in regulatory regions rather than coding regions. Such knowledge is crucial to understand the role of transcriptional levels and gene copy numbers in disease progression or drug efficacy.

## Perspectives and Final Remarks

During the last decades, the zebrafish has proven a valuable and reliable model for basic and applied research in genetics. The recent advent of the CRISPR/Cas9 technology has further enhanced the use of this model system by providing a tool to obtain robust results in functional genomics in a reduced time. At the same time, it has enormously expanded the range of applications for which the zebrafish model can be used. In this review, we have discussed the CRISPR/Cas9-based methodologies developed in zebrafish in the last years and suggest how they can be applied to make more effective the drug discovery process, through faster target validation, more robust disease modeling and more efficacious drug screenings.

**FIGURE 1:**
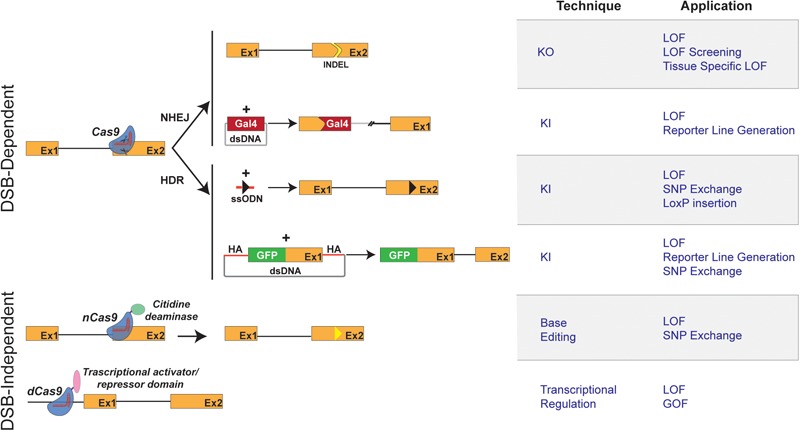
Schematic representation of CRISPR/Cas9 techniques and applications in zebrafish. **(Left)** Graphical representation of CRISPR/Cas9-based methods available for genome engineering in zebrafish. Double strand break (DSB)-Dependent techniques: the Cas9 endonuclease/sgRNA complex induces DSB at the target genomic locus. The NHEJ (non-homologous end joining) DNA repair mechanism leads to the appearance of insertion/deletion (INDELs) which can result in a shift of the open reading frame of the targeted genomic locus, thereby causing gene inactivation (knockout: KO). The targeted insertion (knockin: KI) of donor DNA sequences coding for reporter genes (e.g., Gal 4 transcriptional *trans*-activator) is, in most reports, mediated by NHEJ. The Homology Directed Repair mechanism allows the precise integration of exogenous DNA at a chosen genomic locus. Single-stranded oligodeoxynucleotide (ssODN) or Double stranded vectors harboring homology arms (HA) can be used as donor DNA for the KI of point mutations and reporter genes. Double strand break (DSB)-independent techniques: modified versions of the Cas9 protein, not displaying endonuclease activity, are used. The fusion of a cytidine deaminase to a Cas9 nickase (nCas9) allows a DSB-independent irreversible conversion of one targeted base to another (Base Editing and introduction of Single Nucleotide Polymorphisms, SNPs). Dead Cas9 (dCas9), when fused to a repressor domain or activator domain, can interact to either activate or repress transcription. **(Right)** Table describing the applications of CRISPR/Cas9-based methods depicted in the left panel.

To illustrate our views, we have introduced the current methodologies for generating KOs and KIs and discussed their technical challenges and purposes (**Figure 1**). Besides the technical description, we have presented examples of studies, which, by taking advantage of the combination of the CRISPR/Cas9 system and the zebrafish model, have led to the identification of new therapeutic candidates. As some technical limitations are solved, it is expected that the number of such examples will multiply in the future. To this end, and regardless of the methodology used, we propose below some applications that have the potential to expand the range of CRISPR-based applications of the zebrafish model in the research for therapeutic alternatives to treat human disease.

### High-Throughput Genetic Screens

As previously mentioned, an advantage of the zebrafish model is the possibility of addressing specific phenotypes resulting from gene disruption in a short time and on a large number of animals. In that regard, phenotypic screens can be performed for genes involved in different human pathologies. Genetic hearing loss, for example, can be assessed on a functional level by hearing response assays, but also on a structural level, since the cellular components of the inner ear are highly conserved between humans and zebrafish (Abbas and Whitfield, 2010). Another example would be represented by screens for target identification in cardiomyopathies, as the zebrafish heart physiology is highly analogous to the human (Bakkers, 2011). Importantly, the zebrafish heart at 5dpf is fully functional and readily accessible by non-invasive *in vivo* imaging. High-throughput genetic screens could be advantageous also in cancer research, since it is known that some mutations are only lethal when synergizing with other mutations, a concept known as synthetic lethality (O’Neil et al., 2017). By using CRISPR for performing LOF screens for essential genes in survival, one could identify, in an unbiased manner, conditional lethal genes unique to a specific cancer type, together with genes that are synthetically lethal after somatic mutations or compound treatment. Those genes would bring light into cancer specific vulnerabilities using an *in vivo* model. Therefore, they would be potential targets for drug discovery and/or combinatorial therapy.

### Drug-Target Interaction

For several drugs, some of them already on the market, the domain of interaction of the target protein with the drug is often unknown. A deeper knowledge of drug-target interaction is crucial to design more efficient and less toxic analogs of a given compound. The CRISPR/Cas9 system can be used to determine interacting domains by selecting sgRNAs targeting specific protein regions (Shi et al., 2015). This approach could be applied in zebrafish for analyzing essential domains of any target protein, especially multi-domain proteins, in the context of an *in vivo* assay. Such strategy, coupled with phenotypic analysis of mutant fish, could provide more complex readouts than those provided by *in vitro* systems.

### Humanized Zebrafish Models

A limitation of all model organisms is that, even in cases of high homology with humans, they are sometimes not readily translatable to human biology. *In vitro* models of induced pluripotent stem (iPS) cells or more reliable patient derived iPS cellular systems can overcome such limitation. Nevertheless, recreating *in vitro* the conditions of the native environment of a cellular type is an extremely challenging task. Generating humanized zebrafish can represent a step forward in that desired translatability.

Here, we consider a humanized model to be either a zebrafish transgenic line in which the endogenous gene is substituted with its human ortholog, or a setup in which human tumor cells are xenotransplanted into zebrafish to assess their proliferation or metastatic progression. Xenotransplantation models based on the engraftment of labeled human cancer cells in zebrafish larvae are already established and are used as an alternative to rodent models for drug screening (Fior et al., 2017). However, it is often not clear how specific mutations in cancer cells affect oncologic progression. It would be interesting to use CRISPR/Cas9 in this experimental set up to mutate specific human genes in tumor cells, transplant them in zebrafish and finally analyze the effect of induced loss-of-function in tumor growth and dissemination. Conversely, disruption of genes in the zebrafish host followed by xenotransplantation of human tumor cells would provide insights into a potential involvement on the microenvironment leading to cancer progression and resistance to therapy.

On the other hand, replacement of zebrafish genes with the human ortholog is still not a common and standardized procedure. CRISPR/Cas9 will certainly facilitate the transition toward humanized zebrafish. In fact, both HDR and NHEJ knockin strategies could allow the simultaneous disruption of fish genomic loci and replacement with its human ortholog. That would open interesting avenues in the study of drug-target interaction, if functional full-length human genes were exchanged in the fish genome, or toward personalized medicine, if the human ortholog gene carries specific SNPs related to patient stratification.

### Final Remarks

Lack of efficacy is the major drug attrition cause during clinical development. To moderate future failures, biomedical research requires innovative approaches to identify the right drug targets upfront, understand their role in disease biology and perform preclinical target validation studies in relevant models of human disease. In this review, we have suggested that using zebrafish can help achieving those goals. Furthermore, we have proposed that the advantages obtained by the implementation of CRISPR/Cas9 in zebrafish will have an even deeper impact in the discovery of next generation therapies and treatment paradigms. Now, the use of CRISPR/Cas9 in zebrafish permits: (i) to streamline the identification of disease-relevant targets, and (ii) to build complicated genetic models, which might be key for performing disease-relevant phenotypic drug screenings. Hence, using zebrafish might allow exploiting simultaneously target and phenotypic drug screening strategies, which could result in more successful pipelines at a lower cost and time. The rationale is to narrow down a library of molecules, through *in silico* or *in vitro* methods, against a target identified or validated through a phenotypic drug screening performed in zebrafish. Then, it would be possible to test the efficacy of selected molecules, and possible chemical analogs, on relevant zebrafish disease models through their impact on the pathologic phenotype. Moreover, drug toxicity can be evaluated simultaneously with drug efficacy, providing an early assessment of safety liabilities. Given the low cost and time to perform such a combined screening strategy, it could be possible to test hundreds of molecule-target interactions in a disease-relevant model before entering expensive preclinical regulatory phases. The ultimate goal of such strategy would be to use the unique properties offered by CRISPR/Cas9 to develop humanized zebrafish used in personalized medicine, so each patient will be treated with the drug/set of drugs that are going to be most effective for them.

## Author Contributions

All authors listed have made a substantial, direct and intellectual contribution to the work, and approved it for publication.

## Disclaimer

This work reflects only the author’s view and that the Agency is not responsible for any use that may be made of the information it contains.

## Conflict of Interest Statement

All authors are currently employed by ZeClinics SL.
